# Transcriptional Modulation of Plant Defense Genes by a Bipartite Begomovirus Promotes the Performance of Its Whitefly Vector

**DOI:** 10.3390/v16111654

**Published:** 2024-10-23

**Authors:** Wen-Ze He, Shu-Sheng Liu, Li-Long Pan

**Affiliations:** 1 Ministry of Agriculture Key Lab of Molecular Biology of Crop Pathogens and Insects, Zhejiang Key Laboratory of Biology and Ecological Regulation of Crop Pathogens and Insects, Institute of Insect Sciences, Zhejiang University, Hangzhou 310058, China; hewenze@zafu.edu.cn (W.-Z.H.); shshliu@zju.edu.cn (S.-S.L.); 2 The Rural Development Academy, Zhejiang University, Hangzhou 310058, China

**Keywords:** *Bemisia tabaci*, Sri Lankan cassava mosaic virus, *Nicotiana tabacum*, insect vector–virus–plant tripartite interactions, transcriptome

## Abstract

The majority of plant viruses rely on insect vectors for inter-plant transmission. Amid virus transmission, vector-borne viruses such as begomoviruses may significantly modulate host plants in various ways and, in turn, plant palatability to insect vectors. While many case studies on monopartite begomoviruses are available, bipartite begomoviruses are understudied. More importantly, detailed elucidation of the molecular mechanisms involved is limited. Here, we report the mechanisms by which an emerging bipartite begomovirus, the Sri Lankan cassava mosaic virus (SLCMV), modulates plant defenses against whitefly. SLCMV infection of tobacco (*Nicotiana tabacum*) plants significantly downregulated defenses against whitefly, as whitefly survival and fecundity increased significantly on virus-infected plants when compared to the controls. We then profiled SLCMV-induced transcriptomic changes in plants and identified a repertoire of differentially expressed genes (DEGs). GO enrichment analysis of DEGs demonstrated that the term defense response was significantly enriched. Functional analysis of DEGs associated with defense response revealed that four downregulated DEGs, including *putative late blight resistance protein homolog R1B-17* (*R1B-17*), *polygalacturonase inhibitor-like* (*PGI*), *serine/threonine protein kinase CDL1-like* (*CDL1*), and *Systemin B*, directly contributed to plant defenses against whitefly. Taken together, our findings elucidate the role of novel plant factors involved in the modulation of plant defenses against whitefly by a bipartite begomovirus and shed new light on insect vector–virus–host plant tripartite interactions.

## 1. Introduction

Plant viral pathogens cause widespread crop diseases in agricultural ecosystems [1]. While the inter-plant spread of viruses can be achieved via multiple routes, transmission by insect vectors is the most common. Hemipteran insects such as whiteflies, aphids, and leafhoppers are major groups of insect vectors that transmit over half of the currently known vector-borne plant viruses [1,2]. For virus transmission, the vectors need to feed first on infected plants to acquire the virus and then translocate to uninfected plants to inoculate the virus [2]. During this acquisition–transmission cycle, active interactions may occur among insect vectors, viruses, and plants. For example, viruses may significantly modulate the physiology of host plants and, in turn, the population dynamics of insect vectors [3,4]. As changes in vector population dynamics translate into altered virus spread, such a plant-mediated interaction between viruses and insect vectors serves as one of the major determinants of insect vector population and virus spread dynamics. Due to the recognized importance of these tripartite interactions, a myriad of research efforts have been invested in this fascinating area of research. It has been found that some vector-borne plant viruses may significantly modulate the performance of insect vectors on plants by targeting plant hormonal pathways [5]. Notably, however, detailed elucidation of the mechanisms associated with the modulation of plant resistance against insects is achieved only in a handful of cases. More explorations in this regard will help to advance our understanding of the factors dictating insect vector–virus–plant tripartite interactions.

In recent decades, begomoviruses (family *Geminiviridae*) have emerged as important pathogens in the production of many crops, including tomato, cotton, and cassava [6]. Depending on the number of genomic molecules, begomoviruses can be monopartite or bipartite. Monopartite begomoviruses such as tomato yellow leaf curl virus and tomato yellow leaf curl China virus contain only one genomic molecule. Some monopartite begomoviruses, such as tomato yellow leaf curl China virus, are frequently associated with satellites, including alphasatellites and betasatellites [7]. Bipartite begomoviruses, such as the Sri Lankan cassava mosaic virus (SLCMV), contain two genomic molecules designated as DNA-A and DNA-B [7]. Under natural conditions, begomoviruses are transmitted by whiteflies of the *Bemisia tabaci* complex [8]. While many case studies examining the modulation of whitefly–plant interactions by monopartite begomoviruses are available, bipartite begomoviruses are understudied [5,9]. More importantly, the viral and plant factors involved in the modulation of plant defenses against whitefly by begomoviruses have been investigated only for a few monopartite viruses [3,4,5,9]. Considering the vast number of begomoviral species (https://ictv.global/report/chapter/geminiviridae/geminiviridae/begomovirus, accessed on 29 February 2024), more mechanistic explorations are warranted using previously unexamined begomoviruses such as bipartite ones.

SLCMV is a bipartite begomovirus that causes widespread cassava mosaic disease in Asian countries [10]. Since its first outbreak in Cambodia, SLCMV has rapidly spread to many Asian countries, including Vietnam, China, Laos, and Thailand [11,12,13,14,15]. SLCMV is highly pathogenic to cassava plants, inducing severe leaf curl and mosaic symptoms [10]. Additionally, SLCMV may readily infect Solanaceous plants such as tobacco (*Nicotiana tabacum*) and *Nicotiana benthamiana* [16,17]. As for insect vectors, explorations on SLCMV transmission revealed that different whitefly species transmit SLCMV with disparate efficiencies, and Asia II 1 is the most efficient vector [17]. However, whether and how SLCMV modulates plant–whitefly interactions remain unexplored.

In this study, we first examined the effects of SLCMV infection of tobacco plants on the performance of Asia II 1 whiteflies. Next, we profiled the transcriptomic changes in tobacco plants induced by SLCMV infection. We then identified the differentially expressed genes (DEGs) associated with plant defenses against whitefly and performed functional analysis. Our data add to our knowledge of the whitefly–begomovirus–plant tripartite interactions.

## 2. Materials and Methods

### 2.1. Plants and Insects

Cotton (*Gossypium hirsutum* L. cv. Zhemian 1793) and tobacco (*Nicotiana tabacum* L. cv. NC89) plants were used. Plants were cultured in insect-proof greenhouses at 25 ± 3 °C under natural lighting. Cotton plants were grown to 9–11 true-leaf stage and then used for whitefly rearing. Tobacco plants were grown to the 3–4 true-leaf stage and then used for agro-inoculation. For whiteflies, a culture of Asia II 1 whiteflies, originally collected in field, were maintained on cotton plants. The mt*COI* GenBank accession number of the whitefly culture is DQ309077. The whiteflies were reared in insect-proof cages in climate chambers from 25 to 27 °C, 14:10 light/dark (light: 6:00–20:00) and 60–80% relative humidity. Every 2–3 months, around 50 whiteflies from the culture were subjected to mt*COI* PCR-restriction fragment length polymorphism and sequencing to monitor the purity of whitefly culture [18].

### 2.2. Viruses and Virus-Infected Plants

**Figure 1:**
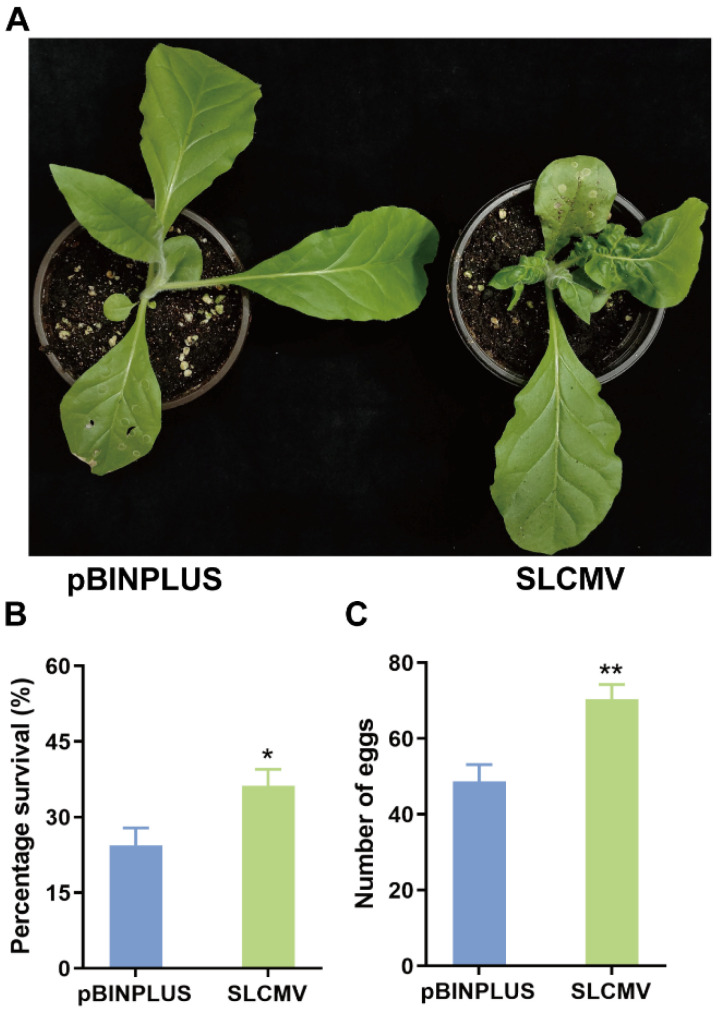
Effect of SLCMV infection of tobacco plants on plant phenotype and whitefly performance. (**A**) picture of tobacco plants. Tobacco plants were inoculated with pBINPLUS (empty vector, control) or SLCMV DNA-A+DNA-B. At 25 days post inoculation, plants showing typical symptoms were used for photographing. (**B**,**C**) survival and fecundity of Asia II 1 whiteflies on tobacco plants. Ten Asia II 1 whiteflies were released into leaf-clip cages that were placed on tobacco leaves. Whitefly survival and fecundity were recorded seven days post whitefly release. N = 27 for B and C. * *p* < 0.05 and ** *p* < 0.01 (independent *t*-test).

An isolate of SLCMV that was characterized before in our laboratory was used [17]. The GenBank accession codes were KT861468 for DNA-A and KT861469 for DNA-B. Agrobacteria containing infectious clones of DNA-A and DNA-B were cultured separately until OD600 reached 1.5–2.0. Agrobacteria were then pelleted and resuspended in resuspension buffer (10 mM MgCl_2_, 10 mM MES, 200 µM acetosyringone). Resuspended agrobacteria solutions containing infectious clones of DNA-A and DNA-B were mixed, and the final OD600 value was 1.0 for both kinds of agrobacteria. Tobacco plants at 3–4 true-leaf stage (about 4 weeks post sowing) were used for agro-inoculation using 1 mL syringes. Control plants were inoculated with agrobacteria containing pBINPLUS (empty vector). About 25 days later, tobacco plants at 7–8 true-leaf stage were used for whitefly bioassay after determining the virus infection status. Symptom inspection (Figure 1A) and PCR detection of SLCMV DNA using primers SLCMV-F and SLCMV-R (Table 1) were conducted to determine the virus infection status.

### 2.3. Whitefly Bioassay

Whiteflies at 0–4 days post-emergence were collected for the bioassay. To enclose whiteflies on plant leaves, leaf-clip cages were used [19]. For each plant, two or three cages were used, one on the second apical fully expanded leaf and one or two on the third. The number of plants tested was 7–11. In total, 10 whiteflies (5 males and 5 females) were introduced into each cage. The plants used for the bioassay were placed in insect-proof cages in the artificial climate chambers mentioned above. The number of live whiteflies and eggs deposited (some may have developed into nymphs) was determined seven days later.

### 2.4. mRNA Library Construction and Sequencing

At 25 days post inoculation, the second apical fully expanded leaves of tobacco plants were sampled and stored in a −80 °C fridge until use. Leaves from three plants were mixed into one sample, and three samples were analyzed for both pBINPLUS and SLCMV treatments. Total RNAs were isolated using TRIzol reagent (Invitrogen, Waltham, MA, USA) following the user’s manual. RNA quality and quantity were determined using NanoDrop ND-1000 (NanoDrop, Waltham, MA, USA). RNA integrity was assessed using Bioanalyzer 2100 (Agilent, Waltham, MA, USA) with RIN number > 7.0 and confirmed using electrophoresis with denaturing agarose gel. Poly(A) RNAs were purified from 1 μg total RNAs using Dynabeads Oligo (dT)25-61005 (Thermo Fisher, Waltham, MA, USA) with two rounds of purification. The poly(A) RNAs were fragmented using Magnesium RNA Fragmentation Module (NEB, Ipswich, MA, USA) at 94 °C for 5–7 min. The cleaved RNA fragments were reverse-transcribed to create the cDNA using SuperScript^TM^ II Reverse Transcriptase (Invitrogen, USA). cDNAs were then used to synthase U-labeled second-stranded DNAs with *Escherichia coli* DNA polymerase I (NEB, USA), RNase H (NEB, USA), and dUTP Solution (Thermo Fisher, USA). An A-base was then added to the blunt ends of each strand, preparing them for ligation to the indexed adapters. Each adapter contains a T-base overhang for ligating the adapter to the A-tailed fragmented DNA. Single- or dual-index adapters were ligated to the fragments, and size selection was performed with AMPureXP beads. After the heat-labile UDG enzyme (NEB, USA) treatment of the U-labeled second-stranded DNAs, the ligated products were amplified with PCR with the following conditions: initial denaturation at 95 °C for 3 min; 8 cycles of denaturation at 98 °C for 15 s, annealing at 60 °C for 15 s, and extension at 72 °C for 30 s; and then final extension at 72 °C for 5 min. The average insert size for the final cDNA library was 300 ± 50 bp. Finally, we performed the 2 × 150 bp paired-end sequencing (PE150) using an Illumina Novaseq^TM^ 6000 (LC-Bio Technology Co., Ltd., Hangzhou, China) following the vendor’s recommended protocol.

### 2.5. Sequencing Data Analysis

Cutadapt software (version 1) (https://cutadapt.readthedocs.io/en/stable/, version:cutadapt-1.9, accessed on 10 June 2021) was used to remove the reads that contained adaptor contamination (command line: ~cutadapt -a ADAPT1 -A ADAPT2 -o out1.fastq -p out2.fastq in1.fastq in2.fastq -O 5 -m 100). After the removal of low-quality and undetermined bases, we used HISAT2 software (version 2.2.1) (https://daehwankimlab.github.io/hisat2/, version:hisat2-2.0.4, accessed on 10 June 2021) to map reads to the genome (*Nicotiana tabacum* Ensembl v96) (command line: ~hisat2 -1 R1.fastq.gz -2 R1.fastq.gz -S sample_mapped.sam). The mapped reads of each sample were assembled using StringTie (version 5.4.6) (http://ccb.jhu.edu/software/stringtie/, version:stringtie-1.3.4d.Linux_x86_64, accessed on 15 June 2021) with default parameters (command line: ~stringtie -p 4 -G genome.gtf -o output.gtf -l sample input.bam). Next, all transcriptomes from all samples were merged to reconstruct a comprehensive transcriptome using gffcompare software (version 1) (http://ccb.jhu.edu/software/stringtie/gffcompare.shtml, version:gffcompare-0.9.8.Linux_x86_64, accessed on 16 June 2021). When the final transcriptome was generated, StringTie and ballgown (version 2.35.0) (http://www.bioconductor.org/packages/release/bioc/html/ballgown.html, accessed on 16 June 2021) were used to estimate the expression levels of all transcripts and perform expression level for mRNAs by calculating FPKM (FPKM = [total exon fragments/mapped reads(millions) × exon length (kB)]) (command line: ~stringtie -e -B -p 4 -G merged.gtf -o samples.gtf samples.bam). The differentially expressed mRNAs were selected with fold change > 2 or fold change < 0.5 and *p* < 0.05 by R package dgeR (version 1) (https://bioconductor.org/packages/release/bioc/html/edgeR.html, accessed on 16 June 2021) or DESeq2 (version 1.44.0) (http://www.bioconductor.org/packages/release/bioc/html/DESeq2.html, accessed on 16 June 2021). Finally, differentially expressed genes (DEGs) were subjected to GO and KEGG enrichment analysis on websites http://geneontology.org and http://www.kegg.jp/kegg (accessed on 16 June 2021).

### 2.6. Analysis of Gene Expression Level Using qPCR

For the analysis of gene expression levels using qPCR, at 25 days post inoculation, the second apical fully expanded leaves were sampled. Total RNAs were isolated using TRIzol reagent (Invitrogen, USA). cDNA synthesis was conducted using an Evo M-MLV RT Kit with gDNA Clean for qPCR (Accurate Biology, Changsha, China). qPCR was performed using the SYBR Green Premix Pro Taq HS qPCR Kit (Accurate Biology, China) and CFX96 Real-Time PCR Detection System (Bio-Rad, Boulder, CO, USA) with the primers listed in Table S1. *GADPH* was used as the housekeeping gene.

### 2.7. Virus-Induced Gene Silencing (VIGS)

For VIGS of tobacco genes, around 300 bp of the coding sequence was cloned into the plasmid pBIN2mDNA1 [20] using the primers listed in Table S1. Recombinant plasmids were mobilized into *Agrobacterium tumefaciens* EHA105 by electro-transformation. Empty pBIN2mDNA1 was used as a control. Agrobacteria containing recombinant or empty pBIN2mDNA1 were cultured and resuspended in the resuspension buffer mentioned above to an OD600 of 2.0. Infectious clones of tobacco curly shoot virus isolate Y35 were cultured and resuspended similarly. Equal amounts of the pBIN2mDNA1 and Y35 cells were mixed and inoculated into tobacco plants (3–4 true leaf stage) using 1 mL syringes. pBIN2mDNA1-*Su*+Y35 served as the positive control. Plants were sampled to determine gene silencing efficiency using qPCR three weeks post inoculation. At four weeks post inoculation, these plants were used for the whitefly bioassay.

### 2.8. Statistical Analysis

Data of the whitefly survival rate were arcsine square root transformed prior to statistical analysis. qPCR data of gene expression level were normalized to plant *GADPH* using the 2^−ΔCt^ method. Comparisons of whitefly survival rate, egg number, and gene expression level were conducted using Student’s independent *t*-test. To clearly illustrate the differences, the data of gene expression level in each of the experiments were normalized to that of the control. All data were presented as the mean ± standard errors of the mean (mean ± SEM), and differences were considered significant when *p* < 0.05. All statistical analyses were conducted using SPSS Statistics 21.0 and EXCEL.

## 3. Results

### 3.1. Effects of SLCMV Infection of Tobacco Plants on Phenotype and Whitefly Performance

At 25 days post inoculation, PCR verified the presence of SLCMV in SLCMV-inoculated tobacco plants (Figure S1). Compared to the control (pBINPLUS), SLCMV-infected tobacco plants exhibited stunted growth, severe leaf curl, and wrinkling (Figure 1A). The following whitefly bioassay showed that SLCMV infection of tobacco plants significantly increased the survival and fecundity of Asia II 1 whiteflies (Figure 1B,C). These results suggest that SLCMV significantly modulates plant defenses against its whitefly vector.

### 3.2. Transcriptome Sequencing, Assembly and Mapping

Three biological replicates were performed for both pBINPLUS- and SLCMV-infected plants. A total of 44.38 Gb of raw data was obtained. After the removal of low-quality bases and undetermined bases, 42.77 Gb of clean data was obtained. The Q20 and Q30 values were 99.98–99.99% and 98.26–99.29%, respectively; the GC content was 43.00–44.50% (Table 1). The mapping rates for the assembled transcriptomes were more than 96.17%, and the ratios of unique mapped reads were more than 76.68% (Table 1).

### 3.3. Gene Expression Analysis

**Figure 2:**
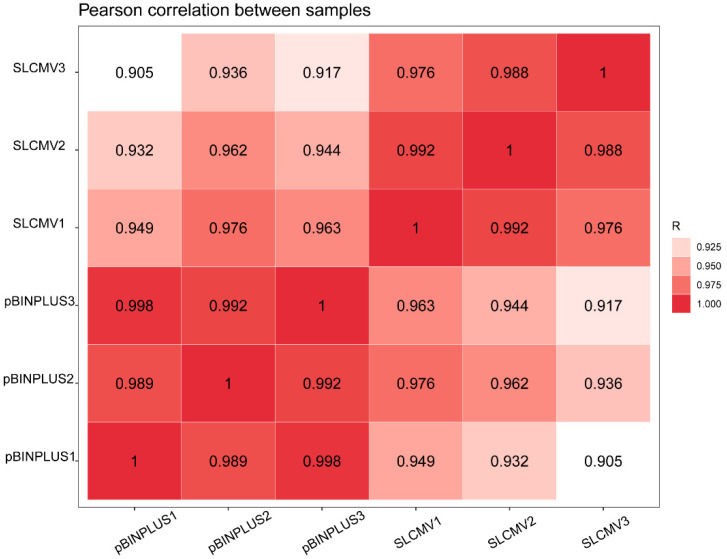
Pearson’s correlation coefficients of overall gene expression patterns between samples. The coefficient values are presented and indicated by the red color.

**Figure 3:**
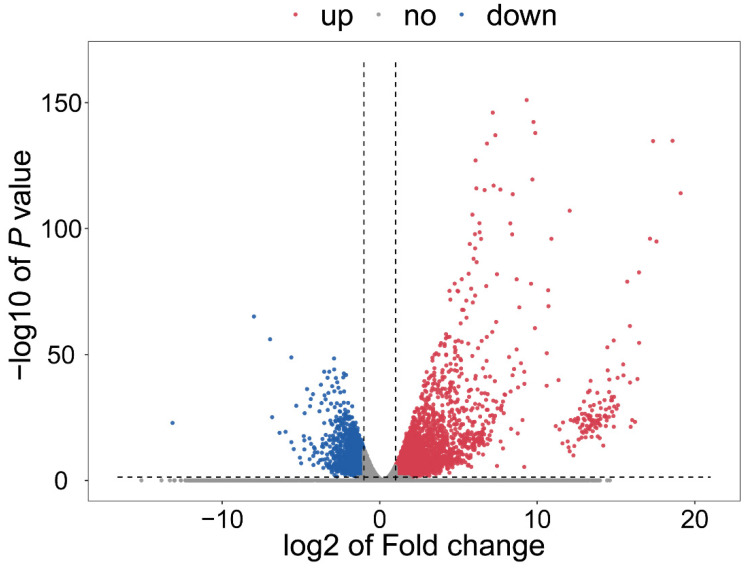
Volcano plot of differentially expressed genes in SLCMV vs. pBINPLUS. The *x*-axis represents the log fold change, and the *y*-axis represents the log significance (*p* value). Blue dots represent downregulated genes, and red dots represent upregulated genes.

The expression level of all genes was calculated (Table S2). Analysis of overall gene expression patterns showed that the Pearson’s correlation coefficients between samples from the same treatment (pBINPLUS or SLCMV) were much higher than those between samples from different treatments (Figure 2). When analyzing differentially expressed genes (DEGs) according to the criteria fold change > 2 or <0.5 and *p* < 0.05, 5278 upregulated and 2134 downregulated DEGs were identified (Table S3 and Figure 3).

### 3.4. Functional Enrichment Analysis of DEGs

**Figure 4:**
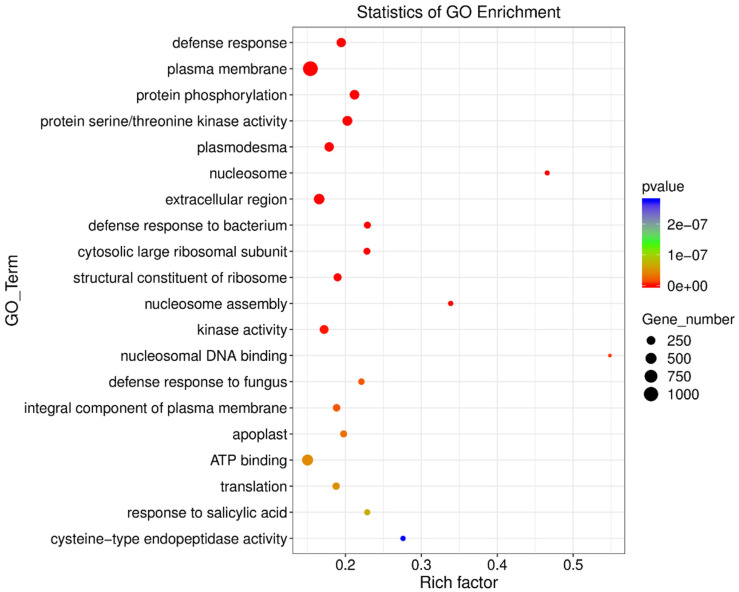
Distribution of the top twenty GO terms in the GO database. The *Y*-axis represents the name of the GO term, and the *X*-axis indicates the rich factor. The *p* value was indicated by the color of the dots, and the number of genes in each term was indicated by the size of the dots.

To identify the DEGs that play pivotal roles in biological pathways and functional networks, a GO enrichment analysis was conducted. DEGs were found to actively participate in various pathways, and the top 20 most significantly enriched GO terms were identified (Table S4 and Figure 4). Of the GO terms, the defense response stood out as the most significantly enriched. Notably, two related terms were similarly enriched, namely the defense response to bacterium and the defense response to fungus. Several terms involved in regulating cellular components were enriched, including the plasma membrane, plasmodesma, nucleosome, extracellular region, and the cytosolic large ribosomal subunit, an integral component of the plasma membrane and apoplast. Additionally, a handful of terms displaying molecular function were enriched, such as protein serine/threonine kinase activity, structural constituent of ribosome, kinase activity, nucleosomal DNA binding, ATP binding, and cysteine-type endopeptidase activity.

### 3.5. Validation of the Expression of Downregulated DEGs in the GO Term Defense Response 

**Figure 5:**
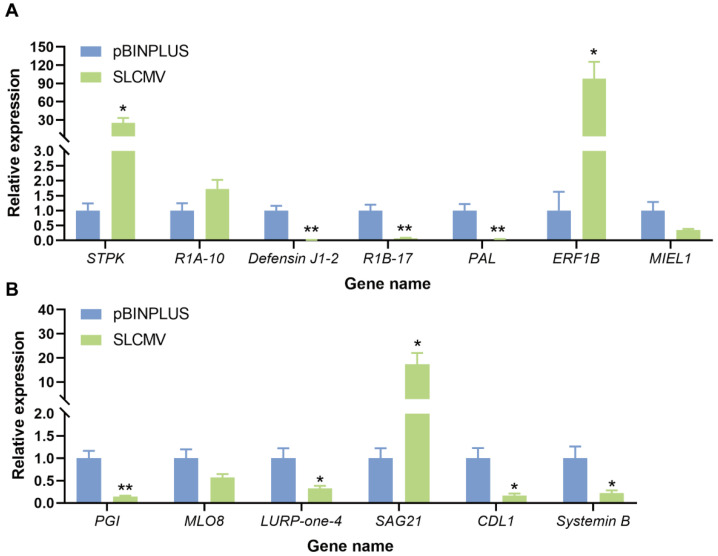
Effect of SLCMV infection of tobacco plants on the expression of DEGs. (**A**,**B**) expression of DEGs. Tobacco plants were inoculated with pBINPLUS (empty vector) and SLCMV DNA-A+DNA-B. Plants were sampled for gene expression analysis at 25 days post inoculation. The number of replicates was 5–6. * *p* < 0.05 and ** *p* < 0.01 (independent *t*-test).

The decreases in plant resistance to whitefly induced by SLCMV may be attributable to the downregulation of defense genes. We thus summarized the downregulated DEGs in the top one enriched GO terms defense responses. In total, twenty DEGs were identified (Table 2). Thirteen DEGs were subjected to qPCR analysis as some DEGs were homologs. In agreement with the transcriptomic data, the expression of seven genes, including *Defensin J1-2*, *R1B-17*, *PAL*, *PGI*, *LURP-one-4*, *CDL1*, and *Systemin B*, was significantly downregulated by SLCMV (Figure 5). However, the expression of three DEGs, including *STPK*, *ERF1B*, and *SAG21*, was significantly induced by SLCMV. Additionally, SLCMV infection of plants did not significantly impact the expression of *R1A-10*, *MIEL1*, and *MLO8*.

### 3.6. Effects of DEG Silencing in Plants on Whitefly Performance

**Figure 6:**
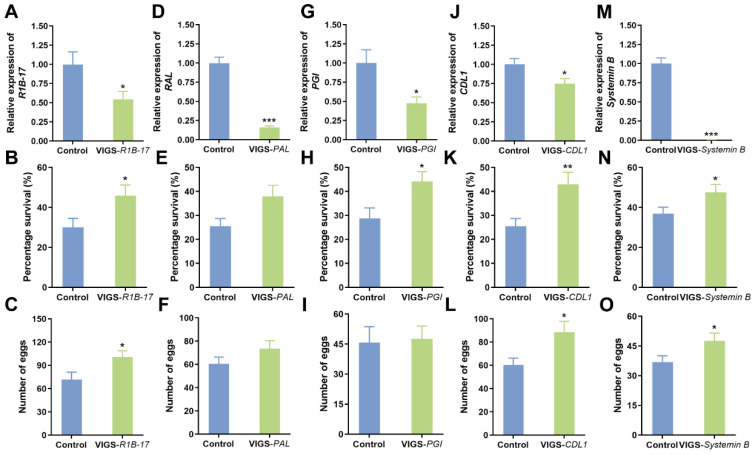
Effects of DEG silencing on whitefly performance. (**A**,**D**,**G**,**J**,**M**) Silencing efficiency; (**B**,**E**,**H**,**K**,**N**) survival rate of whiteflies on control and virus-induced gene silencing (VIGS) plants; (**C**,**F**,**I**,**L**,**O**) fecundity of whiteflies on control and VIGS plants. The number of replicates was 7–19 for (**A**,**D**,**G**,**J**,**M**) and 22–31 for (**B**,**C**,**E**,**F**,**H**,**I**,**K**,**L**,**N**,**O**). * *p* < 0.05, ** *p* < 0.01, and *** *p* < 0.001 (independent *t*-test).

To explore the role of downregulated DEGs in plant defenses against whitefly, we used VIGS coupled with a whitefly bioassay. Analysis of gene silencing efficiency showed that the expression of *Defensin J1-2* and *LURP-one-4* could not be downregulated using our VIGS systems. We thus focused on *R1B-17*, *PAL*, *PGI*, *CDL1*, and *Systemin B*. All five DEGs can be effectively silenced (Figure 6A,D,G,J,M). The whitefly bioassay revealed that the silencing of *R1B-17*, *CDL1*, and *Systemin B* in tobacco plants significantly increased whitefly survival and fecundity (Figure 6B,C,K,L,N,O). The silencing of *PGI* significantly increased whitefly survival but did not impact whitefly fecundity (Figure 6H,I). The silencing of *PAL*, however, did not significantly affect whitefly survival or fecundity (Figure 6E,F).

## 4. Discussions

In this study, we first found that SLCMV infection of tobacco plants significantly increased whitefly performance (Figure 1). We next profiled the transcriptomic changes in tobacco plants induced by SLCMV infection (Table 1 and Figure 2 and Figure 3). Further, we performed GO enrichment analysis and identified DEGs that may be associated with the modulation of plant resistance against whitefly (Table 2 and Figure 4). Finally, we functionally characterized these DEGs and found that SLCMV infection downregulated the expression of some DEGs that positively regulated plant defenses against whitefly (Figure 5 and Figure 6).

While the modulation of plant–whitefly interactions has been explored extensively, most case studies focus on monopartite begomoviruses such as tomato yellow leaf curl China virus [9]. In contrast, bipartite begomoviruses are understudied. More importantly, detailed molecular mechanisms governing the modulation of plant–whitefly interactions by begomoviruses have only been characterized for monopartite begomoviruses. Specifically, a handful of viral and plant factors have been found when investigating tomato yellow leaf curl China virus and its associated betasatellite [3,21,22,23,24]. Additionally, the mechanisms involved in the modulation of plant–whitefly interactions by cotton leaf curl Multan virus (and its associated betasatellite) and tomato yellow leaf curl virus have been uncovered [4,24,25]. Here, we examined the modulation of plant interactions with whitefly by emerging bipartite begomoviruses SLCMV and found that this virus significantly increased whitefly performance on plants. Furthermore, we identified several plant genes that are involved in the modulation of plant defenses against whitefly by SLCMV. Our findings add to the case studies of whitefly–begomovirus–plant tripartite interactions and our knowledge of the molecular mechanisms involved.

In this study, we found that SLCMV infection of tobacco plants significantly increased whitefly survival and fecundity. Additionally, SLCMV downregulated the expression of several plant genes, four of which, namely *R1B-17*, *PGI*, *CDL1*, and *Systemin B*, negatively regulated whitefly performance in plants. *R1B-17* is a member of the late blight resistance R1 gene family that confers resistance to *Phytophthora infestans* in plants [26]. *PGI* encodes a polygalacturonase-inhibiting protein that putatively binds and inhibits the activity of fungal galacturonase polygalacturonase, thereby conferring resistance to fungi in plants [27]. *CDL1* is a serine/threonine protein kinase that regulates the biological function of substrate protein by phosphorylation at serine or threonine sites [28]. While three kinds of genes have been implicated in modulating plant resistance to pathogens [26,29,30], how these genes impact plant–insect interactions remains unknown. Here, we found that *R1B-17*, *PGI*, and *CDL1* positively regulated plant defenses against whitefly, adding to our knowledge of the biological function of these genes and plant resistance genes against whitefly. Moreover, previous studies have revealed the convergence between plant responses against whitefly and pathogens, such as the activation of the salicylic acid signaling pathway [31,32,33]. The findings that three putative anti-pathogen genes also confer resistance against whitefly add further weight to this claim and suggest that resistance genes against whitefly may be recovered from studies on plant–pathogen interactions.

Systemin is a peptide hormone that regulates systemic wound response in plants by modulating the biosynthesis of jasmonates [34]. The fact that *Systemin B* directly contributes to plant defenses against whitefly may be attributable to the jasmonates signaling pathway, as it is the major pathway that controls plant defenses against whitefly [5,33]. In a recent study from our laboratory, *Systemin B* was shown to positively regulate the tobacco jasmonates signaling pathway [25]. Interestingly, βC1 encoded by betasatellite associated with cotton leaf curl Multan virus interfered with the processing of Systemin by a protease, thereby impairing plant defenses against whitefly [25]. In this study, we found that SLCMV may downregulate the expression of *Systemin B*, thereby decreasing plant defenses against whitefly. These findings suggest that *Systemin B* is targeted by multiple begomoviruses for the suppression of plant defenses against whitefly.

Taken together, here we have determined the modulation of plant–whitefly interactions by an emerging bipartite begomovirus. Additionally, we functionally characterized the role of some plant genes using RNA-seq coupled with whitefly bioassay. Our findings expand the case studies of whitefly–begomovirus–plant tripartite interactions and add to our knowledge of the molecular mechanisms involved in these interactions.

## Figures and Tables

**Table 1:** Quality control and mapping of transcriptome data.

Sample	Q20%	Q30%	GC Content%	Valid Reads	Mapped Reads (Ratio)	Unique Mapped Reads (Ratio)
pBINPLUS1	99.98	98.39	43.50	46663582	44889542 (96.20%)	35780224 (76.68%)
pBINPLUS2	99.98	98.26	43.00	48122814	46280054 (96.17%)	37012910 (76.91%)
pBINPLUS3	99.99	99.26	43.00	53348740	51808314 (97.11%)	43440256 (81.43%)
SLCMV1	99.99	99.21	43.00	49338578	47519338 (96.31%)	40136110 (81.35%)
SLCMV2	99.99	99.29	43.00	51991338	49973397 (96.12%)	42327585 (81.41%)
SLCMV3	99.99	99.28	44.50	35685108	34317261 (96.17%)	28506167 (79.88%)

**Table 2:** Downregulated DEGs in the GO term defense response in RNA-seq.

DEG Description	Abbreviation	NCBI Accession Code	Fold Change (SLCMV vs. pBINPLUS)
G-type lectin S-receptor-like serine/threonine protein kinase At4g27290	STPK	XM_016613226.1	0.05
Putative late blight resistance protein homolog R1A-10	R1A-10	XM_016600847.1	0.08
Defensin J1-2-like	Defensin J1-2	XM_016595188.1	0.11
Putative late blight resistance protein homolog R1B-17	R1B-17	XM_016658352.1	0.17
Phenylalanine ammonia-lyase-like	PAL	XM_016625506.1	0.18
Putative late blight resistance protein homolog R1A-4		XP_016512895.1	0.19
Defensin-like protein 1		XM_016621003.1	0.21
Polygalacturonase inhibitor-like		XM_016650945.1	0.24
Ethylene-responsive transcription factor 1B-like	ERF1B	XM_016660099.1	0.34
E3 ubiquitin–protein ligase MIEL1-like	MIEL1	XM_016641417.1	0.35
Phenylalanine ammonia-lyase-like		XM_016646785.1	0.38
Polygalacturonase inhibitor-like	PGI	XM_016650938.1	0.39
MLO-like protein 8	MLO8	XM_016589174.1	0.43
Protein LURP-one-related 4-like	LURP-one-4	XM_016597233.1	0.44
G-type lectin S-receptor-like serine/threonine protein kinase At4g27290 isoform X1		XP_016581305.1	0.44
Protein SENESCENCE-ASSOCIATED GENE 21	SAG21	XM_016583266.1	0.44
Ethylene-responsive transcription factor 1B-like		XM_016620862.1	0.45
Serine/threonine protein kinase CDL1-like		XM_016653465.1	0.47
Serine/threonine protein kinase CDL1-like isoform X2	CDL1	XM_016650790.1	0.48
Hydroxyproline-rich Systemin B	Systemin B	XM_016610586.1	0.48

## Data Availability

The original contributions presented in the study are included in the article/Supplementary Material, further inquiries can be directed to the corresponding author.

## Supplementary Materials

The following supporting information can be downloaded at: https://www.mdpi.com/article/10.3390/v16111654/s1, Figure S1: PCR detection of SLCMV DNA-A in pBINPLUS-inoculated and SLCMV DNA-A+DNA-B inoculated plants; Table S1: Primers used in this study; Table S2: Expression level of all genes; Table S3: List of differentially-expressed genes; Table S4: Results of GO enrichment analysis.

## Availability of Data and Materials

The datasets supporting the conclusions of this article are included within the article and its additional files. The raw data of RNA-seq were deposited in the sequence Read Archive database under the project number PRJNA1138492.
