# Supplementary material for: Transcriptional Modulation of Plant Defense Genes by a Bipartite Begomovirus Promotes the Performance of Its Whitefly Vector
Source: Viruses. 2024 Oct 23;16(11):1654. doi: 10.3390/v16111654 (PMC11598951; doi:10.3390/v16111654)
Supplement: Supplementary file 1 [file viruses-16-01654-s001.zip › viruses-3269906-supplementary/Figure S1.pdf]

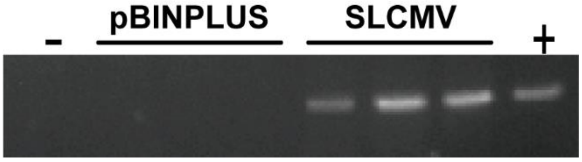

Fig. S1 PCR detection of SLCMV DNA-A in pBINPLUS-inoculated and SLCMV-inoculated plants. At 25 days post inoculation, the first fully-expanded leaves were harvested and subjected to the detection of SLCMV DNA-A. – and + stand for negative control (water) and positive (agrobacteria), respectively.
